# Etiology and Diagnostic Predictors of Acute Undifferentiated Febrile Illness in Adult Hospitalized Patients at a Tertiary Care Center

**DOI:** 10.7759/cureus.106452

**Published:** 2026-04-05

**Authors:** Bharatsing D Rathod, Srikant S Tareekeri, Onkar Awadhiya, Nilesh Kamble, Vishal Shete, Rajashree S Khot, Meena Mishra, Sunita Kumbhalkar

**Affiliations:** 1 General Medicine, All India Institute of Medical Sciences, Nagpur, Nagpur, IND; 2 Microbiology, All India Institute of Medical Sciences, Nagpur, Nagpur, IND

**Keywords:** acute febrile illness, aufi, dengue, enteric fever, leptospirosis, malaria, scrub typhus, tropical fever

## Abstract

Background

Acute undifferentiated febrile illness (AUFI) presents major diagnostic and therapeutic challenges in tropical regions, particularly in resource-limited settings. We prospectively evaluated the etiology, clinical characteristics, diagnostic predictors, and outcomes of AUFI among adult patients admitted to a tertiary care center in Central India.

Methodology

We conducted a prospective observational study of consecutive adult patients hospitalized with AUFI between May 2024 and September 2025. All patients underwent standardized clinical evaluation and routine laboratory testing, including leukocyte counts, platelet counts, liver enzymes (alanine aminotransferase, aspartate aminotransferase), and C-reactive protein (CRP). Targeted diagnostic assays were performed for dengue, malaria, leptospirosis, enteric fever, and scrub typhus. Univariate analyses were used to identify clinical and laboratory predictors of specific etiologies, and effect measures were reported where applicable.

Results

Of the 262 AUFI cases, an etiologic diagnosis was not established in 112 (42.7%) cases. Among diagnosed cases, scrub typhus, dengue, enteric fever, leptospirosis, and malaria were the leading causes. Cases clustered among rural residents (181, 69.1%) and during the monsoon season (188, 71.8% between August and October). Elevated CRP (>50 mg/L) was significantly more frequent in bacterial and rickettsial infections than in viral illness (malaria, 5 (100%); leptospirosis, 14 (77.8%); scrub typhus, 32 (62.7%) vs. dengue, 8 (16.7%). Hepatosplenomegaly was strongly associated with malaria (3 (60%); odds ratio (OR) = 10.1) and was more common in enteric fever (6 (31.6%); OR = 3.29).

Conclusions

In this cohort of hospitalized adults with AUFI, a substantial proportion remained undiagnosed, contributing disproportionately to mortality. Certain clinical signs (such as eschar and hepatosplenomegaly) and routine laboratory markers (particularly CRP) provided valuable discriminatory power, helping to distinguish bacterial and rickettsial infections from viral causes. These findings support the integration of pragmatic clinical and biomarker-based algorithms into empiric management strategies, especially in resource-constrained tropical settings.

## Introduction

Acute undifferentiated febrile illness (AUFI) is defined as fever ≥38°C (≥100.4°F) of less than two weeks’ duration, without an identifiable cause after thorough clinical and laboratory evaluation [1]. It typically presents with abrupt fever, chills, headache, myalgia, fatigue, and malaise. The illness may resolve spontaneously or persist for up to two weeks. AUFI is a major cause of hospitalization in tropical regions and contributes substantially to morbidity and mortality.

The etiologic spectrum is broad, including viral, bacterial, protozoal, and rickettsial pathogens, with prevalence varying by geography and season [1-3]. Historically, malaria, scrub typhus, dengue, enteric fever, and leptospirosis have been predominant in India [4-7]. More recent data, however, indicate a shift toward viral and rickettsial infections, with declining malaria incidence and re‑emergence of scrub typhus and arboviral diseases such as dengue and chikungunya [5-7].

AUFI often presents as a syndromic illness. Fever with rash, myalgia, arthralgia, hemorrhagic manifestations, or jaundice may each reflect multiple etiologies. Laboratory findings, including leukopenia or leukocytosis, thrombocytopenia, abnormal liver enzymes, and elevated C‑reactive protein (CRP), are nonspecific, complicating definitive diagnosis.

Clinical severity ranges from mild, self‑limiting illness to life‑threatening organ dysfunction. In tropical regions, simple diagnostic tools such as peripheral smear or rapid tests for malaria and dengue are widely used, whereas advanced assays (e.g., polymerase chain reaction (PCR) for paramyxoviruses, microscopic agglutination test (MAT) or enzyme-linked immunosorbent assay (ELISA) for leptospirosis, ELISA for rickettsial infections) are required for other pathogens [8,9]. Despite these modalities, studies suggest that 25-50% of AUFI cases in Asia remain undiagnosed [3,10].

Diagnostic accuracy is limited by overlapping clinical features and restricted access to specialized tests. Clinicians often rely on basic laboratory parameters and focused clinical findings to guide empiric therapy, though the performance of these markers has not been systematically defined in endemic settings.

To address these gaps, we conducted a prospective observational study of adults admitted with AUFI to a tertiary care center in Central India. Our objectives were to delineate the etiologic spectrum, characterize clinical and seasonal patterns, and identify predictors that may facilitate early diagnosis and improve targeted management in resource‑constrained environments.

## Materials and methods

Study design and setting

We conducted a prospective, observational study in the Department of General Medicine at All India Institute of Medical Sciences (AIIMS), Nagpur, between May 2024 and November 2025. Consecutive adult patients (≥18 years) admitted with an AUFI of 3-14 days’ duration were enrolled. Patients with immunocompromised states (including HIV infection, hematological malignancies, or use of immunosuppressive therapy), autoimmune diseases, or fever with a localizing focus (such as urinary tract or respiratory tract infection) were excluded.

Definition of acute undifferentiated febrile illness

AUFI was defined as an acute febrile illness characterized by an abrupt onset of fever (≥38°C or ≥100.4°F) lasting ≤14 days, without evidence of localized infection. Localization was excluded through history, physical examination, complete blood count, chemistry profile, urinalysis, and chest radiography at the time of initial presentation [1].

Diagnostic criteria

The definitive diagnosis criteria for specific etiologies of AUFI are shown in Table 1.

**Table 1:** Diagnostic criteria for the specific etiology of AUFI. AUFI = acute undifferentiated febrile illness; ELISA = enzyme-linked immunosorbent assay; RT-PCR = reverse transcription polymerase chain reaction

Specific etiology	Diagnostic criteria
Scrub typhus	Presence of eschar with positive IgM ELISA, or IgM ELISA positivity alone
Dengue	Compatible clinical features with positive dengue IgM or NS1 antigen by ELISA
Malaria	Visualization of trophozoites of *Plasmodium falciparum*, *Plasmodium vivax*, or both on peripheral blood smears
Enteric fever	Positive blood culture for *Salmonella typhi* or *Salmonella paratyphi*, or positive Widal
Leptospirosis	Positive Leptospira IgM ELISA
Chikungunya	Positive IgM ELISA or RT-PCR for chikungunya
Unclear diagnosis	No definitive etiology identified despite a complete evaluation

Sample size

Sample size was calculated using OpenEpi, based on a prior study reporting a 35.9% prevalence of scrub typhus among AUFI cases [4]. Assuming 90% power, α of 0.10, and a 90% confidence interval, the required sample size was 249.

Data collection

After obtaining written informed consent, eligible patients were enrolled consecutively. Demographic and clinical data were recorded in a standardized case record form, and comorbidities were documented. Routine investigations included complete blood count, peripheral smear for malaria parasites, liver and renal function tests, CRP, urine analysis, chest radiography, and abdominal ultrasonography.

Etiology-specific tests comprised leptospira IgM, scrub typhus IgM, chikungunya IgM, reverse transcription polymerase chain reaction (RT-PCR) for chikungunya, Widal test, blood culture, and dengue NS1 and IgM ELISA. For acute-phase illness (≤5 days after onset), dengue NS1 ELISA was performed; for late-phase illness (>5 days after onset), dengue IgM ELISA (J. Mitra & Co. Pvt. Ltd.) was used. Scrub typhus IgM ELISA and leptospira IgM ELISA (J. Mitra & Co. Pvt. Ltd.) were performed on or after the seventh day of fever.

A single blood culture was obtained from all patients using an aerobic BacT/Alert 3D (bioMérieux) bottle and incubated for up to five days in the BacT/Alert system. A positive Widal test, defined as a titer >100 for O (somatic) and >200 for H (flagellar) antigens of *Salmonella typhi* in a single serum sample during the second or third week of illness, was considered indicative of recent infection.

Investigations were repeated as clinically indicated to monitor leukocyte count, platelet count, and CRP trends. Patients were followed throughout hospitalization, and outcomes, including complications, recovery, or mortality, were documented.

Statistical analysis

Data were analyzed using SPSS version 26.0 (IBM Corp., Armonk, NY, USA). Continuous variables were expressed as mean ± standard deviation and compared using independent t-tests or one-way analysis of variance (ANOVA), as appropriate. Categorical variables were summarized as frequencies and percentages, and compared using the chi-square or Fisher’s exact test.

Univariate analysis was performed to identify clinical and laboratory predictors of specific etiologies. Variables with p <0.10 were entered into multivariate binary logistic regression models using forward stepwise selection. Adjusted odds ratios (ORs) with 95% confidence intervals (CIs) were calculated. Clinical prediction scores for scrub typhus and dengue were derived by assigning points proportional to adjusted ORs. A two-tailed p-value <0.05 was considered statistically significant.

Ethical considerations

The study was approved by the Institutional Ethics Committee of AIIMS Nagpur (approval number: IEC/Pharmac/2024/861, dated May 17, 2024). Written informed consent was obtained from all participants. Patient confidentiality was strictly maintained, and participants were informed of their right to withdraw at any time without impact on ongoing treatment.

## Results

Study population and etiologic distribution

**Figure 1:**
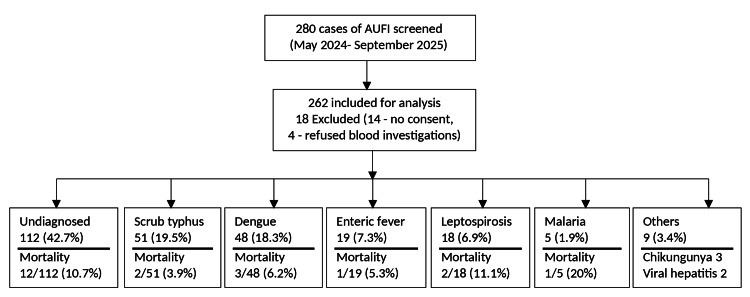
Flowchart of AUFI cases. Flowchart depicting the diagnostic outcomes and mortality rates among 262 patients with AUFI screened between May 2024 and September 2025. AUFI = acute undifferentiated febrile illness

A total of 262 patients with AUFI were enrolled (Figure 1). Demographic and clinical characteristics are summarized in Table 2. Most patients were young adults, with nearly two-thirds between 21 and 50 years of age. Tropical infections accounted for more than half of the cases, with scrub typhus and dengue being the most frequent. Despite extensive microbiological evaluation, over two-fifths of patients remained undiagnosed, underscoring the diagnostic challenges inherent to AUFI.

**Table 2:** Baseline demographic and clinical characteristics of AUFI cases (n = 253*). *: Out of the total 262 cases, we have taken 253 for further analysis, which were cases belonging to major etiologies of AUFI (viz., dengue, scrub, leptospirosis, enteric fever, malaria, and undiagnosed AUFI). **: One-way ANOVA (F-statistic) was used for comparing continuous variables across multiple groups. ^†^: Chi-square test (χ²) was used for categorical variables with adequate expected cell frequencies when assumptions were met (expected cell counts ≥5). ^‡^: Fisher’s exact test was used for categorical variables when expected cell count was <5 in more than 20% of the cells. ***: Adventitious breath sounds/abnormal breathing. AUFI = acute undifferentiated febrile illness; ANOVA = analysis of variance

Variable	Scrub typhus (n = 51)	Dengue (n = 48)	Malaria (n = 5)	Enteric fever (n = 19)	Leptospirosis (n = 18)	Undiagnosed (n = 112)	Test statistic	P-value
Age (years), mean ± SD	44.5 ± 17.9	29.4 ± 10.8	35.2 ± 11.5	29.8 ± 12.1	38.2 ± 14.8	38.8 ± 16.5	F = 5.23**	<0.001
Male, n (%)	31 (60.8%)	24 (50.0%)	3 (60.0%)	11 (57.9%)	11 (61.1%)	62 (55.4%)	χ² = 1.56^†^	0.756
Rural, n (%)	31 (60.8%)	15 (31.2%)	5 (100.0%)	6 (31.6%)	12 (66.7%)	67 (59.8%)	Fisher’s^‡^	<0.001
Hospital stay (days), mean ± SD	7.3 ± 3.0	6.0 ± 3.8	13.4 ± 9.8	8.2 ± 6.8	9.1 ± 4.8	6.3 ± 3.5	F = 4.87**	<0.001
Duration of fever (days), mean ± SD	8.5 ± 3.7	5.6 ± 2.5	10.8 ± 4.5	7.2 ± 3.8	10.1 ± 5.3	6.1 ± 3.2	F = 6.45**	<0.001
Tachycardia (>100 beats/minute), n (%)	17 (33.3%)	14 (29.2%)	3 (60.0%)	7 (36.8%)	6 (33.3%)	42 (37.5%)	χ² = 18.67^†^	0.002
Relative bradycardia, n (%)	1 (2.0%)	1 (2.1%)	0 (0.0%)	7 (36.8%)	1 (5.6%)	5 (4.5%)	Fisher’s^‡^	<0.001
Hypotension, n (%)	4 (7.8%)	3 (6.2%)	3 (60.0%)	1 (5.3%)	1 (5.6%)	8 (7.1%)	Fisher’s^‡^	<0.001
Tachypnea (>20/minute), n (%)	16 (31.4%)	10 (20.8%)	4 (80.0%)	5 (26.3%)	4 (22.2%)	30 (26.8%)	χ²=14.89†	0.012
Hypoxia (SpO₂ <94%), n (%)	16 (31.4%)	6 (12.5%)	5 (100.0%)	5 (26.3%)	3 (16.7%)	23 (20.5%)	Fisher’s^‡^	<0.001
Altered sensorium, n (%)	7 (13.7%)	0 (0.0%)	3 (60.0%)	0 (0.0%)	0 (0.0%)	13 (11.6%)	Fisher’s^‡^	<0.001
Pallor, n (%)	25 (49.0%)	12 (25.0%)	5 (100.0%)	11 (57.9%)	12 (66.7%)	54 (48.2%)	Fisher’s^‡^	<0.001
Icterus, n (%)	13 (25.5%)	1 (2.1%)	0 (0.0%)	4 (21.1%)	6 (33.3%)	10 (8.9%)	Fisher’s^‡^	<0.001
Edema feet, n (%)	9 (17.6%)	3 (6.2%)	1 (20.0%)	1 (5.3%)	3 (16.7%)	12 (10.7%)	Fisher’s^‡^	0.354
Hepatomegaly, n (%)	8 (15.7%)	3 (6.2%)	3 (60.0%)	8 (42.1%)	4 (22.2%)	20 (17.9%)	Fisher’s^‡^	<0.001
Splenomegaly, n (%)	5 (9.8%)	2 (4.2%)	3 (60.0%)	7 (36.8%)	3 (16.7%)	13 (11.6%)	Fisher’s^‡^	<0.001
Hepatosplenomegaly, n (%)	3 (5.9%)	1 (2.1%)	3 (60.0%)	6 (31.6%)	2 (11.1%)	10 (8.9%)	Fisher’s^‡^	<0.001
Respiratory abnormality^***^, n (%)	23 (45.1%)	8 (16.7%)	4 (80.0%)	5 (26.3%)	3 (16.7%)	30 (26.8%)	χ² = 19.34^†^	<0.001
Abdominal tenderness/distension, n (%)	8 (15.7%)	8 (16.7%)	2 (40.0%)	6 (31.6%)	4 (22.2%)	23 (20.5%)	Fisher’s^‡^	0.507

Seasonality and geographic distribution

Cases clustered during the monsoon and immediate post-monsoon months, with August contributing disproportionately. Scrub typhus and dengue demonstrated overlapping peaks in late monsoon, whereas leptospirosis was concentrated earlier in the season. Malaria was confined to August, while enteric fever showed a distinct pre-monsoon pattern. These observations highlight the strong seasonal drivers of AUFI and the differing temporal profiles of individual etiologies.

Clinical features

Mean fever duration varied significantly across etiologies (Table 2). Headache, myalgia, and gastrointestinal symptoms were the most frequent manifestations overall. Certain features were more etiology-specific: breathlessness was prominent in scrub typhus, altered sensorium in malaria and scrub typhus, and bleeding in severe dengue and malaria. Distinctive vital sign abnormalities provided potential bedside clues: relative bradycardia in enteric fever, hypotension and tachypnea in malaria, and tachypnea in scrub typhus.

Laboratory findings

Laboratory abnormalities differed by etiology (Table 3). Severe anemia was most often observed in leptospirosis and malaria. Dengue was characterized by leukopenia, whereas scrub typhus and leptospirosis more frequently showed leukocytosis. Elevated CRP was nearly universal in malaria and leptospirosis, common in scrub typhus, and less typical in dengue, suggesting differential inflammatory responses across infections.

**Table 3:** Laboratory parameters of AUFI cases (n = 253). *: One-way ANOVA (F-statistic) was used for comparing continuous variables across multiple groups. ^†^: Chi-square test (χ²) was used for categorical variables with adequate expected cell frequencies when assumptions were met (expected cell counts ≥5). ^‡^: Fisher’s exact test was used for categorical variables when expected cell count <5 in more than 20% of the cells. AUFI = acute undifferentiated febrile illness; TLC = total leukocyte count; AST = aspartate aminotransferase; ALT = alanine aminotransferase; CRP = C-reactive protein; ANOVA = analysis of variance

Parameter	Scrub typhus (n = 51)	Dengue (n = 48)	Malaria (n = 5)	Enteric fever (n = 19)	Leptospirosis (n = 18)	Undiagnosed (n = 112)	Test statistic	P-value
Hemoglobin (g/dL), mean ± SD	10.2 ± 2.4	11.1 ± 4.0	7.8 ± 1.2	10.7 ± 3.2	9.1 ± 2.8	10.5 ± 3.1	F = 1.98*	0.089
TLC (cells/mm^3^), mean ± SD	11,000 ± 6,000	9,000 ± 6,000	8,500 ± 3,800	10,200 ± 5,100	11,500 ± 6,800	10,100 ± 5,900	F = 4.23*	<0.001
<4,000, n (%)	8 (15.7%)	15 (31.2%)	0 (0.0%)	2 (10.5%)	3 (16.7%)	20 (17.9%)	Fisher’s^‡^	0.02
>10,000, n (%)	19 (37.3%)	8 (16.7%)	1 (20.0%)	6 (31.6%)	6 (33.3%)	40 (35.7%)	χ² = 7.8^†^	0.16
Platelets (×10³/mm^3^), mean ± SD	54 ± 67	58 ± 71	42 ± 38	148 ± 102	85 ± 85	95 ± 88	F = 5.89*	<0.001
<20,000, n (%)	11 (21.6%)	11 (22.9%)	1 (20.0%)	0 (0.0%)	2 (11.1%)	13 (11.6%)	Fisher’s^‡^	0.03
20,000–50,000, n (%)	9 (17.6%)	12 (25.0%)	2 (40.0%)	1 (5.3%)	4 (22.2%)	20 (17.9%)	Fisher’s^‡^	0.09
>50,000–150,000, n(%)	16 (31.37%)	18 (37.5%)	2 (40%)	6 (31.58%)	7 (38.89%)	42 (37.5%)	χ² = 8.7^†^	0.12
>150,000, n (%)	15 (29.4%)	7 (14.6%)	0 (0.0%)	12 (63.2%)	5 (27.8%)	37 (33.0%)	Fisher’s^‡^	0.011
Serum creatinine (mg/dL), mean ± SD	1.82 ± 1.86	1.73 ± 2.50	4.20 ± 5.15	1.35 ± 1.48	2.05 ± 2.12	1.68 ± 1.95	F = 3.12*	0.008
≥1.2, n (%)	25 (49.0%)	13 (27.1%)	3 (60.0%)	5 (26.3%)	8 (44.4%)	37 (33.0%)	χ² = 19.56^†^	<0.001
Urea (mg/dL), mean ± SD	42.5 ± 38.2	38.6 ± 42.1	92.5 ± 82.3	31.8 ± 27.5	50.2 ± 41.8	40.3 ± 35.6	F = 2.89*	0.012
Total bilirubin (mg/dL), mean ± SD	1.8 ± 2.1	1.2 ± 1.4	3.2 ± 2.6	1.4 ± 1.6	3.1 ± 3.4	1.6 ± 1.8	F = 4.67*	<0.001
Albumin (g/dL), mean ± SD	3.15 ± 0.72	3.61 ± 0.68	2.55 ± 0.78	3.28 ± 0.58	3.02 ± 0.72	3.35 ± 0.68	F = 4.23*	<0.001
<3.5, n (%)	32 (62.7%)	17 (35.4%)	3 (60.0%)	12 (63.2%)	14 (77.8%)	63 (56.2%)	χ² = 20.12^†^	<0.001
AST (U/L), mean ± SD	162 ± 208	116 ± 123	145 ± 98	95 ± 88	520 ± 1,439	128 ± 195	F = 3.78*	0.002
>40, n (%)	42 (82.4%)	41 (85.4%)	4 (80.0%)	12 (63.2%)	13 (72.2%)	80 (71.4%)	χ² = 22.34^†^	<0.001
ALT (U/L), mean ± SD	167 ± 260	100 ± 72	55 ± 18	92 ± 78	441 ± 1,111	115 ± 185	F = 3.23*	0.015
>40, n (%)	40 (78.4%)	41 (85.4%)	3 (60.0%)	14 (73.7%)	14 (77.8%)	77 (68.8%)	χ² = 17.89^†^	0.002
CRP (mg/L), mean ± SD	68.5 ± 42.3	24.8 ± 18.6	105.2 ± 58.4	43.8 ± 27.2	85.4 ± 49.8	52.3 ± 38.5	F = 8.92*	<0.001
>50, n (%)	32 (62.7%)	8 (16.7%)	5 (100.0%)	8 (42.1%)	14 (77.8%)	54 (48.2%)	Fisher’s^‡^	<0.001

Complications and outcomes

Complications are detailed in Table 4. Hospital stay was the longest for malaria, followed by leptospirosis and enteric fever. Overall mortality among patients with acute tropical fevers was 8.3% (21 of 253). Malaria (20.0%, 1 of 5) and leptospirosis (11.1%, 2 of 18) demonstrated the highest case‑fatality rates. Nearly half of all deaths (n = 12) occurred in patients without a definitive etiologic diagnosis (10.7%, 12 of 112), highlighting the considerable burden of undiagnosed AUFI.

**Table 4:** Complications of AUFI (n = 253). ^†^: Chi-square test (χ²) was used for categorical variables with adequate expected cell frequencies. ^‡^: Fisher’s exact test used when expected cell count <5 in more than 20% of the cells. AUFI = acute undifferentiated febrile illness; AKI = acute kidney injury; ARDS = acute respiratory distress syndrome

Complication	Scrub typhus (n = 51)	Dengue (n = 48)	Malaria (n = 5)	Enteric fever (n = 19)	Leptospirosis (n = 18)	Undiagnosed (n = 112)	Test statistic	P-value
AKI	22 (43.1%)	12 (25.0%)	3 (60.0%)	5 (26.3%)	8 (44.4%)	37 (33.0%)	χ² = 24.67^†^	<0.001
Transaminitis	31 (60.8%)	28 (58.3%)	4 (80.0%)	7 (36.8%)	12 (66.7%)	71 (63.4%)	χ² = 18.45^†^	0.002
ARDS	8 (15.7%)	4 (8.3%)	1 (20.0%)	0 (0.0%)	2 (11.1%)	7 (6.2%)	Fisher’s^‡^	<0.001
Hepatic dysfunction	13 (25.5%)	8 (16.7%)	4 (80.0%)	5 (26.3%)	9 (50.0%)	31 (27.7%)	χ² = 21.34^†^	<0.001
Encephalopathy	4 (7.8%)	0 (0.0%)	2 (40.0%)	0 (0.0%)	0 (0.0%)	8 (7.1%)	Fisher’s^‡^	0.002
Shock	3 (5.9%)	2 (4.2%)	2 (40.0%)	1 (5.3%)	1 (5.6%)	8 (7.1%)	Fisher’s^‡^	0.045

Predictors of specific etiologies

Independent predictors of scrub typhus and dengue are presented in Table 5 and Table 6. These highlight distinct clinical and laboratory features that may aid early recognition and guide targeted management.

**Table 5:** Clinical and laboratory parameters as predictors of scrub typhus in AUFI cases (n = 51 vs. 90 other diagnosed). AUFI = acute undifferentiated febrile illness; CRP = C-reactive protein; OR = odds ratio; CI = confidence interval

Variable	Unadjusted OR (95% CI)	Adjusted OR (95% CI)	P-value
Fever duration >7 days	2.15 (1.06-4.36)	1.16 (1.11-1.21)	<0.001
Presence of eschar	28 (54.9%)	—	<0.001
Breathlessness	4.02 (1.75-9.25)	4.96 (3.38-7.30)	<0.001
Hepatosplenomegaly	0.31 (0.09-1.08)	—	NS
Leukocytosis >10,000	1.87 (0.89-3.92)	2.31 (1.64-3.24)	<0.001
Hypoalbuminemia <3.5	1.48 (0.74-2.96)	2.32 (1.68-3.20)	<0.001
CRP >50 mg/L	1.81 (0.90-3.64)	3.45 (2.12-5.61)	<0.001

**Table 6:** Clinical and laboratory parameters as predictors of dengue in AUFI cases (n = 48 vs. 93 other diagnosed). AUFI = acute undifferentiated febrile illness; CRP = C-reactive protein; OR = odds ratio; CI = confidence interval

Variable	Unadjusted OR (95% CI)	Adjusted OR (95% CI)	P-value
Fever duration <7 days	6.10 (2.50-14.9)	0.81 (0.76-0.85)	<0.001
Presence of rash	9.35 (4.11-21.3)	12.8 (5.87-27.9)	<0.001
Absence of breathlessness	13.8 (1.85-103)	8.45 (2.14-33.4)	<0.001
Hepatosplenomegaly	0.11 (0.01-0.83)	—	NS
Platelets <150,000	2.53 (1.04-6.15)	2.09 (1.47-2.98)	<0.001
Leukopenia <4,000	5.24 (2.20-12.5)	2.37 (1.56-3.59)	<0.001
CRP <50 mg/L	2.41 (1.02-5.69)	3.87 (1.78-8.42)	<0.001

## Discussion

In this prospective cohort of hospitalized adults with AUFI, we observed a substantial burden of vector-borne and zoonotic diseases, with clear clustering during specific seasons and in rural regions. These epidemiological patterns carry important implications for public health policy in tropical areas where resources remain limited.

Epidemiological profile and disease burden

The demographic profile of our cohort, with a mean age of 37.5 years and peak incidence in the 21-30-year age group, mirrors findings from prior studies in India and other tropical regions. Abhilash et al., for example, reported a comparable mean age of 37.4 years in a South Indian cohort of 1,258 AUFI patients, with nearly 70% of cases occurring between 21 and 50 years [4]. This age distribution reflects the occupational and environmental exposures of economically active populations engaged in agriculture and outdoor labor, where contact with vectors and reservoirs is frequent. The male predominance in our study (54.6%) further supports this occupational risk profile, as men are more likely to participate in outdoor work associated with heightened exposure.

The rural predominance (69.1%) underscores the critical role of zoonotic and vector-borne transmission in agricultural and peri-urban environments. This finding is particularly relevant in the context of climate change, which is increasingly recognized as a driver of zoonotic disease emergence and transmission [11]. Geographic variation by etiology was also evident: malaria, scrub typhus, and leptospirosis clustered in rural areas, whereas enteric fever and dengue were more common in urban settings. These patterns align with established epidemiological principles: scrub typhus and leptospirosis are classical rural zoonoses linked to mite-infested vegetation and flooding [10,12,13], while enteric fever and dengue thrive in urban environments due to sanitation challenges and the adaptation of *Aedes* mosquitoes to urban habitats [14,15]. The predominance of undiagnosed cases in rural areas strongly suggests the presence of unrecognized zoonotic pathogens, highlighting the urgent need for enhanced diagnostic capacity in rural healthcare systems.

Distribution of causes and diagnostic challenges

Scrub typhus and dengue emerged as the most common etiologies, together accounting for nearly 38% of cases. This distribution reflects current trends in tropical India. Bonell et al., in a systematic review, emphasized the growing recognition of scrub typhus as a major cause of acute febrile illness in South and Southeast Asia, historically underdiagnosed due to limited diagnostic tools and clinical awareness [12]. Similarly, Devasagayam et al. documented a considerable burden of scrub typhus across India, with prevalence ranging widely depending on geography, season, and diagnostic methodology [13].

Malaria accounted for only 1.9% of cases in our study, a marked decline compared with earlier reports. For instance, Joshi et al. (n = 1,197) reported malaria in 12% of AUFI cases in Central India in 2008 [16]. This reduction may reflect the success of vector control programs, improved diagnostic practices that reduce false positives, or genuine changes in transmission dynamics [17]. Nevertheless, the clinical severity of malaria in our cohort, universal hypoxia, frequent altered sensorium, and a 20% mortality rate, underscores its continued potential for life-threatening illness, even at lower incidence.

Prevalence of leptospirosis is known to vary with geography and season, influenced by flooding, agricultural practices, and rodent reservoir density. Russell et al. highlighted the diagnostic challenges of leptospirosis, noting that even in endemic regions, diagnosis is often delayed or missed due to nonspecific clinical features and limited access to confirmatory testing [18]. Jiménez et al. further emphasized that leptospirosis remains underdiagnosed worldwide, particularly in tropical and subtropical regions, where it should be considered in any patient presenting with fever, myalgia, and potential exposure to contaminated water or soil [10].

Burden of undiagnosed acute undifferentiated febrile illness

In our study, 42.7% of AUFI cases remained without a definitive diagnosis despite comprehensive microbiological evaluation, including serological testing for scrub typhus, dengue, leptospirosis, malaria, enteric fever, and chikungunya. This substantial proportion highlights a critical diagnostic gap that extends beyond our setting and reflects a broader global challenge in the management of AUFI. Susilawati et al., in a systematic review of AUFI in Asia, reported undiagnosed case frequencies ranging from 8% to 80% [19]. Similarly, Leelarasamee et al. documented 61.3% undiagnosed cases in a multicentric prospective study of 1,240 AUFI patients in Thailand [20].

The consistency of this finding across diverse contexts suggests systemic shortcomings in contemporary diagnostic methodologies rather than localized inadequacies. Several factors contribute to this gap. First, the serological window period: IgM antibodies typically appear five to seven days after symptom onset, and many of our undiagnosed patients presented early, likely before seroconversion. Second, the limited pathogen panel: our diagnostic algorithm did not include testing for several emerging or under-recognized pathogens endemic to tropical India, such as additional rickettsial species, Q fever, brucellosis, hantavirus, arboviruses beyond dengue, and atypical bacterial pathogens. Third, technical limitations: serological assays often demonstrate imperfect sensitivity and specificity, with cross-reactivity between closely related organisms complicating interpretation.

Clinical profiles of the undiagnosed group provide important clues. Nearly half had CRP levels above 50 mg/L, suggesting possible bacterial or rickettsial infections, as such elevations are uncommon in viral illnesses. The predominantly rural distribution (59.8%) points toward unidentified zoonotic pathogens. Moreover, the mortality rate of 10.7% in this group underscores the clinical significance of the diagnostic gap and the urgent need for improved diagnostic capacity.

Rather than viewing this category as a static endpoint, it should be considered a transitional stage in the diagnostic continuum. In practice, such cases warrant a structured pathway that includes repeat clinical evaluation with attention to atypical or evolving presentations, targeted laboratory testing for uncommon infectious and autoimmune etiologies, and, when appropriate, advanced imaging or referral to specialized centers. By outlining these potential steps, we emphasize that “undiagnosed” AUFI represents an opportunity for systematic reassessment, and future research should focus on refining diagnostic algorithms to minimize this category.

Taken together, these findings emphasize that current diagnostic methods, while effective for common pathogens, fail to capture a large proportion of AUFI etiologies. As Barathan argued, the management of AUFI must “move from fever to action,” with diagnosis, treatment, and prevention strategies firmly grounded in evidence [8]. The challenge lies in developing practical, affordable diagnostic algorithms that can reduce the burden of undiagnosed cases in low-resource tropical settings where AUFI is most prevalent.

Seasonal patterns and vector ecology

Seasonal clustering was a striking feature of our cohort, with 71.8% of cases occurring between August and October, corresponding to the monsoon and immediate post-monsoon period. This pattern strongly suggests vector-borne and waterborne transmission pathways and is consistent with established epidemiological trends in monsoon-endemic regions. The timing of peaks aligns with vector ecology: *Anopheles* mosquitoes breed in monsoon-created habitats, *Aedes* mosquitoes thrive in water-storage containers, and *Leptotrombidium* mites reach peak density in moist scrub vegetation during the monsoon.

Climate change is increasingly recognized as a driver of altered disease seasonality and geographic expansion of vector-borne diseases. Variability in rainfall and temperature influences vector populations, pathogen development rates, and human exposure patterns [11]. Such climate-related factors may explain shifts in transmission dynamics, including the earlier onset of disease activity observed in our study, with nearly 10% of cases occurring in July, likely linked to changes in pre-monsoon rainfall.

Leptospirosis demonstrated distinct monsoon clustering, with two-thirds of cases occurring between July and September. Transmission is closely tied to flooding, which increases human contact with water contaminated by rodent urine and displaces rodent populations into human dwellings. Rodríguez-Vidigal et al. reported similar seasonal clustering in Southwestern Spain [21], while Mishra et al. emphasized the importance of preventive strategies in India, including minimizing exposure to floodwaters, wearing protective footwear, and implementing rodent control measures [22].

Enteric fever, in contrast, exhibited pre-monsoon clustering in May-June. This deviation may reflect hygiene challenges during water scarcity, increased fecal-oral transmission when clean water is limited, and enhanced *Salmonella* survival in food and water under high pre-monsoon temperatures. Particularly concerning was the sharp rise in undiagnosed cases in August, accounting for nearly half of all undiagnosed presentations. This temporal clustering strongly suggests that many of these cases represent unrecognized vector-borne or zoonotic infections associated with monsoon transmission, rather than isolated non-infectious causes of fever.

Clinical presentation and distinguishing characteristics

Our study delineated several clinical and laboratory features with strong discriminatory value across etiologies, providing practical guidance for bedside diagnosis in resource-limited settings where definitive testing may be delayed or unavailable. The presence of eschar in 54.9% of scrub typhus cases (p < 0.001) reinforces its role as a pathognomonic marker. Yet, its absence in nearly half of patients underscores that scrub typhus cannot be excluded solely on clinical grounds.

Previous work by Bonell et al. highlighted the wide variability in eschar detection rates (20-80%), influenced by examination timing, the thoroughness of whole-body inspection, including covered areas, and patient skin tone, which affects visibility [12]. Importantly, eschars often occur in concealed, moist regions such as the axilla, groin, inframammary folds, and genitalia. This distribution emphasizes the need for systematic, comprehensive physical examination, particularly in endemic regions.

Enteric fever was distinguished by the step-ladder fever pattern and relative bradycardia, with adjusted ORs of 18.3 (p < 0.001) and 19.5 (p < 0.001), respectively. Although highly specific, these classical features were observed in only 63.2% and 36.8% of cases, limiting sensitivity. Reliance on textbook descriptions is therefore problematic, as host immunity, prior antibiotic exposure, pathogen virulence, and timing of presentation all modulate clinical expression.

In dengue, rash emerged as a particularly useful diagnostic clue, present in 79.2% of cases (adjusted OR = 22.1, p < 0.001). Typically appearing between days four and seven, the rash evolves from macular erythema to a maculopapular eruption with characteristic islands of sparing. National guidelines emphasize recognition of this rash, alongside biphasic fever and warning signs of severe disease, i.e., plasma leakage, hemorrhage, and organ impairment [23]. Conversely, the rarity of breathlessness in dengue (2.1% vs. 22.4% in other causes, OR = 0.08, p = 0.001) served as a strong negative predictor, helping to distinguish dengue from rickettsial infections, which more frequently involve respiratory compromise.

Hepatosplenomegaly provided further diagnostic specificity, occurring in 60.0% of malaria and 31.6% of enteric fever cases, but rarely in dengue (2.1%) or scrub typhus (5.9%). The underlying mechanisms differ: in malaria, sequestration of parasitized erythrocytes and reticuloendothelial activation drive hepatosplenic enlargement [17], whereas in enteric fever, intracellular *Salmonella* involvement of the reticuloendothelial system is responsible [24]. The adjusted OR of 10.1 (p = 0.012) for malaria underscores the importance of meticulous abdominal examination in patients with AUFI.

C-reactive protein as a distinguishing biomarker

Among laboratory parameters, CRP emerged as a particularly robust discriminator between bacterial/rickettsial and viral etiologies. Elevated CRP (>50 mg/L) was universal in malaria (100%), frequent in leptospirosis (77.8%, OR = 4.23, p = 0.009) and scrub typhus (63.5%, OR = 3.45, p < 0.001), but uncommon in dengue (16.7%, OR = 0.17, p < 0.001). This finding provides a pragmatic decision-making framework: markedly elevated CRP strongly favors bacterial or rickettsial infection, warranting antimicrobial therapy, whereas low CRP suggests viral etiology, most often dengue, where antibiotics are unnecessary and supportive care is paramount.

The mechanistic basis lies in divergent immune responses. Bacterial and rickettsial infections trigger substantial interleukin-6 production, driving hepatic synthesis of acute-phase proteins. In contrast, viral infections such as dengue elicit relatively modest acute-phase responses. Pourzangiabadi et al. noted that dengue pathobiology is characterized by limited acute-phase protein elevation compared with bacterial infections, reflecting distinct immunopathological pathways [15].

Clinical application of CRP-guided therapy in AUFI has important implications for antimicrobial stewardship. By curbing inappropriate antibiotic use in viral infections while ensuring timely treatment of bacterial and rickettsial causes, CRP measurement can enhance both patient outcomes and public health.

Complications and clinical severity

The complication rates observed in our study highlight the considerable morbidity associated with tropical febrile illnesses and identify high-risk groups requiring vigilant monitoring. Acute respiratory distress syndrome (ARDS), acute kidney injury (AKI), and thrombocytopenia emerged as the predominant complications, most strongly linked to malaria, leptospirosis, and scrub typhus. These findings underscore the diverse clinical manifestations of tropical fevers and emphasize the importance of early recognition of syndromic patterns that predict severe disease.

ARDS was the most severe complication, occurring in 8.7% of the overall cohort, though incidence varied substantially by etiology: malaria (20%), scrub typhus (15.7%), leptospirosis (11.1%), and dengue (8.3%). Kumar et al. previously identified scrub typhus as an under-recognized cause of acute febrile illness, frequently complicated by AKI (53%), ARDS (57%), and shock (16%), with significant mortality [25]. The pathophysiology of ARDS differs across infections: in scrub typhus and leptospirosis, direct endothelial injury and inflammatory mediator release drive diffuse alveolar damage and capillary leakage; in malaria, sequestration of parasitized erythrocytes within pulmonary microvasculature precipitates ARDS; and in dengue, plasma leakage and fluid overload exacerbate respiratory compromise. Recognition of dyspnea and hypoxia as critical warning signs is essential, as timely escalation of care and intensive care unit referral can mitigate ARDS-related mortality.

AKI was another major complication, affecting 33% of patients overall, with the highest rates in malaria (60%), leptospirosis (44.4%), and scrub typhus (43.1%). Leptospirosis is particularly associated with severe AKI. Sykes et al. reported that AKI occurs in 40-60% of hospitalized patients with severe leptospirosis (Weil’s disease), with 10-20% requiring dialysis [26]. Mechanisms vary by etiology: leptospirosis induces direct tubular injury through leptospiral toxins and immune-mediated glomerular damage; scrub typhus causes microvascular endothelial injury and acute tubular necrosis; malaria-associated AKI results from sequestration, hemolysis-related hemoglobinuria, and inflammatory injury [10,17,18,25]. Collectively, these complication patterns identify malaria, leptospirosis, and scrub typhus as infections with high potential for multi-organ involvement and mortality, underscoring the importance of early biomarker assessment (including CRP), close physiologic monitoring, and prompt escalation to critical care when indicated.

Thrombocytopenia was nearly ubiquitous, affecting 60.7% of patients overall and exceeding 70% in dengue, scrub typhus, and leptospirosis. Pathogen-specific mechanisms contribute: dengue causes marrow suppression, direct megakaryocyte infection, and immune-mediated platelet destruction; scrub typhus promotes endothelial injury and consumptive coagulopathy; and malaria leads to splenic sequestration, oxidative platelet damage, and immune clearance. Despite frequent severe thrombocytopenia, bleeding complications were uncommon, suggesting that low platelet counts alone rarely precipitate hemorrhage in the absence of plasma leakage or coagulopathy. These findings support conservative transfusion strategies in the absence of active bleeding, consistent with contemporary dengue management guidelines [23].

Mortality in the cohort was substantial at 9.9%, with marked variation across etiologies. Malaria and leptospirosis carried the highest case fatality rates, while scrub typhus mortality was comparatively lower, likely reflecting early empiric doxycycline use and heightened clinical suspicion. Notably, a large proportion of deaths occurred in the undiagnosed group, underscoring the clinical and public health consequences of diagnostic gaps and the potential role of unrecognized or emerging pathogens in driving severe disease.

Limitations

This study has several limitations. Conducted at a single tertiary care center, it may overrepresent severe presentations compared with community-based cohorts. Some etiologic groups were small, limiting the precision of subgroup estimates, particularly for malaria. Convalescent serology was not systematically obtained, potentially reducing diagnostic yield for certain infections. The diagnostic panel did not include multiplex molecular assays for a broader range of pathogens, contributing to the large undiagnosed fraction. Finally, the study period may not capture interannual variation in pathogen circulation driven by climatic or ecological changes.

Strengths

Despite these limitations, the study has notable strengths. The prospective design, consecutive enrollment without loss to follow-up, standardized clinical and laboratory assessment, and systematic outcome ascertainment enhance the reliability of findings. Importantly, this study captured the full clinical spectrum of hospitalized AUFI in a high-burden region, providing robust data on seasonality, geography, biomarkers, complications, and mortality, information directly relevant to clinicians and public health planners.

Implications and future directions

The findings carry immediate clinical and research implications. Clinically, incorporation of CRP into early diagnostic algorithms may help distinguish bacterial/rickettsial from viral etiologies, guiding empiric therapy while preserving antimicrobial stewardship. Public health interventions should prioritize vector control, targeted prevention during the monsoon season, and strengthening of rural diagnostic capacity.

Future research should focus on validating CRP-guided antibiotic algorithms in randomized trials, deploying multiplex molecular diagnostics to reduce the undiagnosed burden, and developing clinical prediction scores for bedside triage. Prospective surveillance is needed to detect shifting epidemiology and emerging pathogens. Additionally, economic evaluations of diagnostic strategies and studies optimizing antimicrobial regimens for tropical febrile illnesses are warranted.

## Conclusions

In this hospital-based cohort of adults with AUFI, scrub typhus and dengue emerged as the most frequent identifiable causes. Yet, a substantial proportion of cases remained undiagnosed, and this group accounted for a disproportionate share of mortality. Malaria, leptospirosis, and scrub typhus were particularly associated with multi-organ complications, including AKI, ARDS, and severe thrombocytopenia, underscoring their potential for rapid clinical deterioration. CRP levels greater than 50 mg/L proved to be a valuable biomarker, reliably distinguishing bacterial and rickettsial infections from viral etiologies. This finding has direct clinical relevance: in settings where definitive diagnostics are unavailable, CRP measurement may guide empiric management, ensuring timely antimicrobial therapy while avoiding unnecessary antibiotic use in viral illnesses such as dengue. The implications extend beyond individual patient care. Strengthening diagnostic capacity, validating biomarker-guided treatment algorithms, and reinforcing seasonal vector control are essential strategies to reduce morbidity and mortality. Equally important is investment in rural healthcare infrastructure, which remains the frontline defense against tropical febrile illnesses. Taken together, these findings highlight both the challenges and opportunities in managing AUFI in resource-constrained tropical settings. By integrating clinical features with pragmatic biomarkers such as CRP, clinicians can improve diagnostic accuracy, optimize therapy, and, ultimately, reduce the burden of severe disease.
